# Comparative metabolism and tolerability of racemic primaquine and its enantiomers in human volunteers during 7-day administration

**DOI:** 10.3389/fphar.2022.1104735

**Published:** 2023-01-16

**Authors:** Washim Khan, Yan-Hong Wang, Narayan D. Chaurasiya, N. P. Dhammika Nanayakkara, H. M. Bandara Herath, Kerri A. Harrison, Gray Dale, Donald A. Stanford, Eric P. Dahl, James D. McChesney, Waseem Gul, Mahmoud A. ElSohly, David Jollow, Babu L. Tekwani, Larry A. Walker

**Affiliations:** ^1^ National Center for Natural Products Research, The University of Mississippi, University, MS, United States; ^2^ Department of Infectious Diseases, Division of Drug Discovery, Southern Research Institute, Birmingham, AL, United States; ^3^ Ironstone Separations Inc., Etta, MS, United States; ^4^ ElSohly Laboratories Inc., Oxford, MS, United States; ^5^ Pharmaceutics and Drug Delivery, School of Pharmacy, The University of Mississippi, University, MS, United States; ^6^ Professor Emeritus, Department Cell and Molecular Pharmacology and Experimental Therapeutics, Medical University of South Carolina, Charleston, SC, United States

**Keywords:** primaquine, enantiomers, racemate, metabolism, clinical pharmacokinetics, safety

## Abstract

Primaquine (PQ) is an 8-aminoquinoline antimalarial, active against dormant *Plasmodium vivax* hypnozoites and *P. falciparum* mature gametocytes. PQ is currently used for *P. vivax* radical cure and prevention of malaria transmission. PQ is a racemic drug and since the metabolism and pharmacology of PQ’s enantiomers have been shown to be divergent, the objectives of this study were to evaluate the comparative tolerability and metabolism of PQ with respect to its two enantiomers in human volunteers in a 7 days’ treatment schedule. Fifteen subjects with normal glucose-6-phosphate dehydrogenase (G6PDn) completed four arms, receiving each of the treatments, once daily for 7 days, in a crossover fashion, with a 7–14 days washout period in between: *R*-(−) enantiomer (RPQ) 22.5 mg; *S*-(+) enantiomer (SPQ) 22.5 mg; racemic PQ (RSPQ) 45 mg, and placebo. Volunteers were monitored for any adverse events (AEs) during the study period. PQ and metabolites were quantified in plasma and red blood cells (RBCs) by UHPLC-UV-MS/MS. Plasma PQ was significantly higher in SPQ treatment group than for RPQ. Carboxy-primaquine, a major plasma metabolite, was much higher in the RPQ treated group than SPQ; primaquine carbamoyl glucuronide, another major plasma metabolite, was derived only from SPQ. The ortho-quinone metabolites were also detected and showed differences for the two enantiomers in a similar pattern to the parent drugs. Both enantiomers and racemic PQ were well tolerated in G6PDn subjects with the 7 days regimen; three subjects showed mild AEs which did not require any intervention or discontinuation of the drug. The most consistent changes in G6PDn subjects were a gradual increase in methemoglobin and bilirubin, but these were not clinically important. However, the bilirubin increase suggests mild progressive damage to a small fraction of red cells. PQ enantiomers were also individually administered to two G6PD deficient (G6PDd) subjects, one heterozygous female and one hemizygous male. These G6PDd subjects showed similar results with the two enantiomers, but the responses in the hemizygous male were more pronounced. These studies suggest that although the metabolism profiles of individual PQ enantiomers are markedly different, they did not show significant differences in the safety and tolerability in G6PDn subjects.

## 1 Introduction

Globally, the number of malaria deaths has dropped by 63% over the past 15 years as a result of improved access to medications and insecticide-treated bed nets (WHO, 2021). As a result of this accomplishment, more challenging objectives have been set, including a vision from the WHO to eradicate malaria within the next 15 years by reducing morbidity and mortality by 90%. This battle will require a robust arsenal of effective antimalarial drugs, as pressures of drug resistance continually undermine the public health effectiveness. In addition, the proportion of malaria cases attributable to the *Plasmodium vivax* parasite is growing in many parts of the world, and this presents additional challenges because of the difficulty in eradicating the dormant hypnozoites in the liver. At present, only the 8-aminoquinoline drug class is effective for these forms of the parasite, which are also formed with *P. ovale*. Though several treatments are available for the asexual blood stages of malaria, still primaquine (PQ) and tafenoquine (TQ) are the only choices for the liver hypnozoites (Baird and Rieckmann, 2003; Baird, 2018). PQ has a broad spectrum of activity against most *Plasmodium* species, also showing efficacy against the late-stage gametocytes in blood.

Although the 8-aminoquinolines are generally very well tolerated, there is a major limitation in their potential to cause hemolysis in patients with glucose-6- phosphate dehydrogenase (G6PD) deficiency (Bancone and Chu, 2021). G6PD deficiency is the most common enzyme deficiency worldwide and is prominent in many malaria-endemic regions (Nkhoma et al., 2009). In these patients, the oxidative stress caused by metabolites of PQ and TQ can cause red cell damage, leading to their removal from the circulation.

Since its introduction in the early 1950s, PQ has been used as a racemic mixture of (*S*)- and (*R*)-enantiomers. Previous studies from our laboratory and others indicate that the stereochemistry of PQ significantly affects the efficacy, metabolism, and toxicity in animal models (Nanayakkara et al., 2014; Saunders et al., 2014; Fasinu et al., 2016). The stereochemistry of other 8-aminoquinolines (as exemplified for NPC1161) demonstrated a significant impact on efficacy and toxicity (Tekwani and Walker, 2006; Nanayakkara et al., 2008). In a mouse model of infection, SPQ showed both increased activity and higher toxicity than RPQ (Nanayakkara et al., 2014). In the Rhesus monkey models the efficacy of both enantiomers was similar (Schmidt et al., 1977; Saunders et al., 2014), but RPQ showed greater hepatotoxicity compared to SPQ or racemate (Schmidt et al., 1977).

In the first study to look at this question in human volunteers, we observed that after the administration of racemic PQ, the two enantiomers are indeed sharply distinct in their primary metabolic pathways and pharmacokinetic profile (Tekwani et al., 2015). The RPQ appears to account for the vast majority of circulating carboxyprimaquine (cPQ), derived from oxidation of the terminal primary amine by monoamine oxidases. Recently we confirmed and extended these studies in healthy, G6PD normal volunteers, by administering the individual enantiomers separately (single oral dose) and comparing their pharmacokinetics and metabolism, along with the racemic version in a crossover fashion. Two major metabolites, cPQ and PQ-N-carbamoyl glucuronide (PQ-N-CG), were profiled in plasma. cPQ was the predominant plasma metabolite with the (*R*)-enantiomer, many fold higher than the parent drug, but PQ-N-CG was not detected. In contrast, for the (*S*)-enantiomer, PQ-N-CG was the major plasma metabolite, while very low levels of cPQ were seen (Khan et al., 2022a). Both enantiomers and the racemate were well tolerated at single doses of 22.5 mg and 45 mg for enantiomers and racemate, respectively.

Typically, the observed hemolytic toxicity of PQ in G6PD deficient subjects is observed only after a few days of administration. Presumably, this is because of the time required to generate sufficient oxidative stress by metabolite redox cycling, secondary intracellular damage and external membrane signaling, and the kinetics of splenic sequestration. Based on the divergent metabolism of PQ enantiomers in humans, and the differential safety profiles in animal studies, the present study explored the safety, tolerability, and metabolism of the individual enantiomers along with racemic PQ, administered with daily dosing for 7 days. 7 days’ dosing of PQ has been suggested to be effective in preventing malaria relapse in people with *P. vivax* (Milligan et al., 2020). The studies were conducted in healthy G6PD normal (G6PDn) volunteers during 7 days administration. We were also able to explore these parameters with multiple doses in two subjects with G6PD deficiency (G6PDd).

## 2 Methodology

### 2.1 Chemicals, reagents, and study materials

**FIGURE 1:**
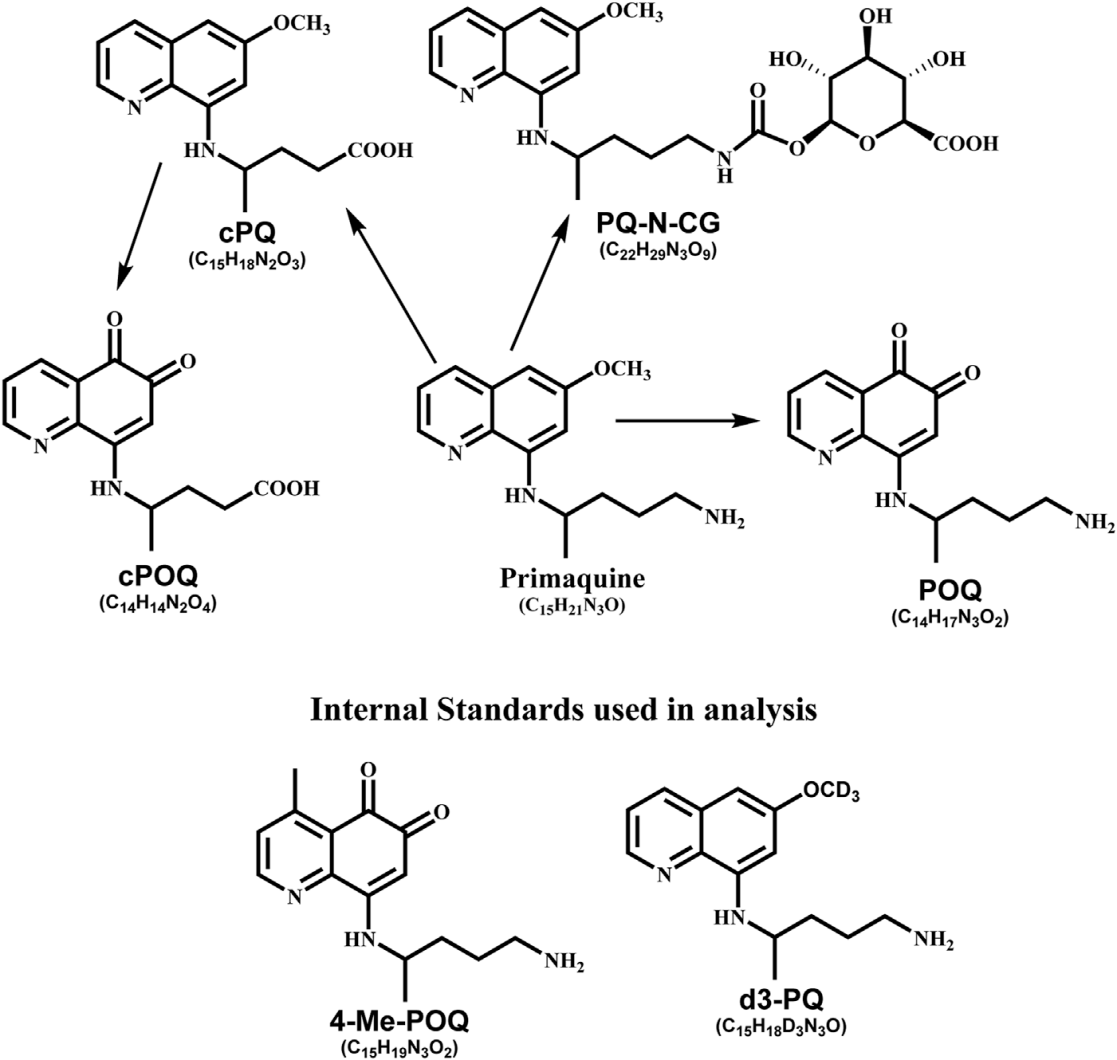
Chemical structure of analyte used in this study.

For analysis, HPLC grade solvents, e.g., methanol, acetonitrile, and formic acid (FA), were purchased from Thermo Scientific (Rockford, IL, United States). Water used for analysis was purified using Millipore Synergy® UV Ultrapure Water Purification System (EMD Millipore Corporation, MA, United States). Reference standards, primaquine diphosphate was purchased from Sigma (St Louis, MO, United States). Different metabolites of PQ and internal standards were synthesized at the National Center for Natural Products Research, University of Mississippi. Synthesis of reference standards have been described in the previous article; primaquine-5,6-orthoquinone (POQ) (Potter et al., 2015), carboxyprimaquine-5,7-orthoquinone (cPOQ) (Khan et al., 2021), PQ-N-CG (Fasinu et al., 2016), cPQ (McChesney and Sarangan, 1984), 4-methylprimaquine-5,6-orthoquinone (4-MePOQ), and deuterated primaquine (d3-PQ) (Khan et al., 2022b). The racemic PQ, PQ enantiomers and placebo capsules used for the clinical study were prepared with complete characterization, purity, and stability, by ElSohly Laboratories, Inc (ELI), Oxford, MS. Each capsule contained the desired specific amount of PQ base as the diphosphate salt. Structure of all analytes are shown in Figure 1.

### 2.2 Clinical study

#### 2.2.1 Study design, ethics, and participants

This study was conducted at the National Center for Natural Products Research, University of Mississippi, MS, United States, in accordance with the ethical principles under protocols approved by the Division of Research Integrity and Compliance-Institutional Review Board, Office of Research and Sponsored Programs, University of Mississippi (IRB# 18–075) and by the Human Research Protection Office of the United States Army Medical Research and Development Command. The ClinicalTrials.gov Identifiers for these studies are NCT03934450 and NCT04073953. A signed written consent was obtained from each individual who participated in this study. Normal healthy adults aged 18–65 years were screened. Screening included history, physical exam, vital signs, electrocardiogram, and blood sampling for baseline hematology, clinical chemistry panel, and G6PD activity. Participants in these trials were healthy, non-smokers, and ranging in age from 20 to 26 years old, with an average body weight of 68 and 80 kg for females and males, respectively. Additionally, eligible female subjects were required not to be pregnant, or breastfeeding, or expected to conceive during the study, or lactating, or of childbearing potential. Volunteers with liver, kidney, hematological, cardiac, and autoimmune disorders were excluded.

Clinical pharmacokinetic studies in G6PDn subjects were single-blinded, placebo-controlled crossover studies conducted at the National Center for Natural Products Research, University of Mississippi, United States of America. Dose selection was based on the clear safety and tolerability from our previous single dose study using SPQ, RPQ, and RSPQ (Khan et al., 2022a) and also from previous clinical studies with racemic PQ (Bancone and Chu, 2021). The study in G6PDn volunteers consisted of four arms. Each subject participated in all four arms, with appropriate washout. The first arm involved the administration of 45 mg of RSPQ once daily for 7 days, followed by at least a 1 week washout; arm two involved administration of 22.5 mg of RPQ every day for 7 days, followed by at least a 1 week washout period; arm three involved administration of placebo every day for 7 days, followed by at least a 1 week washout; and arm four involved administration of 22.5 mg of SPQ every day for 7 days. For power considerations, we used the approach of Kang et al. (2005). Because the key question is whether a reliable clinically important difference in the hematological liability exists (to warrant field trials of the single enantiomer). Assuming we could establish an 80% difference between the two enantiomers in terms of the hematological effects, even allowing an inter-subject variability of 80%, 18 subjects would equate to a power level of 0.8 (*α* = 0.05) (Kang et al., 2005). Thus 18 subjects were enrolled for the study. But only 15 subjects completed all four arms, with complete PQ and metabolite data.

After a light breakfast, one of the PQ doses (RPQ, SPQ, or RSPQ) or placebo was administered to volunteers divided into different groups. Dosing was once daily on days 0–6, for seven consecutive doses. In this study, blood samples (10 mL) were collected by direct venipuncture on day zero pre-dose, on day three pre-dose and at 1 hour post-dose, on day five pre-dose and at 1 hour post dose, and on day seven, 24 h after the last dose. Within 1 h of blood sample collection, it was centrifuged at 15294 *g* for 10 min at 4°C, plasma and blood cells were separated. Packed red cells were washed with cold phosphate buffered saline (PBS), centrifuged, and supernatant discarded. White buffy coat as WBC found in between the layer of RBCs and PBS, were also collected for future studies. The separated plasma and RBC pellets were aliquoted and preserved at −80°C for studies.

Only two volunteers with G6PDd conditions were enrolled in two arms of the multidose pharmacokinetic study of PQ enantiomers. Volunteers were enrolled to receive 15 mg of SPQ every day for 5 days, followed by 2 weeks washout period, then 15 mg of RPQ every day for 4 days. Blood samples were collected at pre-dose and 2 h after oral administration of PQ on days 0, 1, 2, 3, and 4.

#### 2.2.2 Determination of G6PD enzyme activity and biochemical parameters

G6PD status was assessed in each subject by screening prior to enrollment. Hematological and biochemical parameters, including G6PD activity and liver enzyme were assessed by certified clinical laboratory methods by LabCorp (Memphis, TN). For that, blood samples were collected in a different specified vacutainer tube containing with and or without EDTA. Blood samples were sent on the same day of collection. For the determination of G6PD activity, the method biochemical, spectrophotometric method based on the formation of NADPH as estimated by monitoring UV absorbance of the sample at 340 nm.

#### 2.2.3 Adverse events and safety monitoring

Safety was evaluated during the study by monitoring of vital signs (blood pressure, pulse, respiratory rate, oxygen saturation and body temperature), and by recording any adverse events (AEs) at sampling times, and at follow-up visits after study completion. Analysis of biochemical safety and hematological parameters was performed by LabCorp (Memphis, TN). All available data from human volunteers who received either test drug and or placebo, were included in the summaries of the safety data.

### 2.3 Bioanalytical assay for quantification of PQ and its metabolites in plasma and RBCs

All the stored plasma and RBCs samples were thawed in ice and processed to quantify analytes. PQ, cPQ, and PQ-N-CG by a validated ultra-high performance liquid chromatography coupled with Xevo G2-S QToF mass spectrometer (UHPLC-MSMS) (Waters Corp., Milford, MA, United States) (Khan et al., 2022a). As the levels of POQ and cPOQ in plasma were very low, and QToF was not sensitive enough, these two analytes were quantified using Xevo TQ-S triple quadrupole mass spectrometer (UHPLC-TQ-MS, Waters Corp., Milford, MA, United States). The method for quantification of these two analytes was validated as per USFDA guidelines (USFDA, 2018). The method for extraction and analysis of POQ and cPOQ in red blood cells (RBCs) was previously described (Khan et al., 2021). Analysis was carried out using gradient elution and detected by electrospray ionization (ESI) in positive mode. Chromatographic separation was performed using a reversed-phase column (Acquity UPLC HSS T3, 1.8 µm, 2.1 × 100 mm, Waters, United States) set at 35°C. The mobile phase consisted of solvent A (water with 0.05% formic acid) and solvent B (acetonitrile with 0.05% formic acid, v/v). For the analysis of plasma, gradient elution was started with 95% A (0 min), a linear gradient from 95% to 55% A (0–9 min), later up to 0% A (9–10 min). For analysis of RBCs, the gradient elution was started with 15% B at the beginning and increased to 55% B at 3.5 min. To detect analytes, ionization was achieved using electrospray ionization (ESI) operated in positive-ion mode (M + H). The MS/MS transition for POQ, cPOQ, PQ, cPQ, PQ-N-CG, 4-MePOQ and d3-PQ were 260.1 > 175.0, 275.2 > 175.0, 260.1 > 175.1, 275.1 > 175.1, 480.2 > 243.1, 274.2 > 189.0, and 263.2 > 178.1, respectively. All data collected in centroid mode were acquired using MassLynx™ NT 4.1 software. Accurate mass tolerance of molecular ion and major fragments was limited to 5 ppm, while minor fragments of parent ions were tolerated up to 10 ppm.

Blood samples for PQ and its metabolite analysis were drawn at baseline, then on days 3 and 5 pre-dose and at 1 h post-dose, and then on day 7 at 24 h after the last dose. These were analyzed as described above, and the relative plasma exposure of the different enantiomers and racemate were compared, along with the major metabolites.

### 2.4 Statistical analysis

Statistical analysis was performed using the GraphPad prism software version 22.0 (SPSS Inc., Chicago, IL, United States). All data, including PK and biochemical parameters, were summarized using descriptive statistics. Values are expressed as the mean ± SEM for all parameters.

## 3 Results

### 3.1 Safety, tolerability and biochemical profiling assessment of G6PDn subjects

Eighteen G6PDn subjects were enrolled and 15 completed all four arms, receiving RPQ, SPQ, RSPQ, and placebo, separately, in crossover fashion. In each cycle, subjects received one dose of PQ daily for 7 days. In between each cycle, subjects were given at least a 1 week washout period. The baseline demographic and clinical characteristics of the clinical study subjects of both categories are shown in Table 1. The mean age and body weight (±SEM) of G6PDn subjects were 22.0 ± 0.4 years and 168 ± 7 lb, respectively. No changes in BP, pulse, respiratory rate, oxygen saturation, body temperature and weight were observed. The most consistent findings in the G6PDn subjects were elevations in methemoglobin and very small increases in bilirubin, but neither resulted in clinical symptoms, nor required discontinuation of drug.

**TABLE 1:** Subject demographics and baseline clinical characteristics that participated in study.

Parameters	Total	Gender	
		Male	Female
**G6PD normal**	15	11	04
African/Afr. American	01	01	01
Caucasian	10	06	04
Asian	04	04	00
Age (Mean, SD)		22, 1.9	
Age range		20–26	
**G6PD deficient**	02	01	01
African/Afr. American	02	01	01
Age		42	23

No serious adverse events (AEs) or withdrawals due to AEs were observed in G6PDn subjects. The adverse events and their incidence are tabulated in Table 2. Three subjects reported mild AEs. None of these three required withdrawal from the study. Two of the events, in the judgment of the study director and physician, were likely unrelated to the drug treatment. These two showed transient mild elevations in aspartate transaminase (AST) and alanine transaminase (ALT) levels, likely attributable to acute alcohol consumption in one subject (during the SPQ arm) and one with a latent past history of gallbladder trouble (increased AST/ALT during placebo arm). The third subject—who had the most prominent methemoglobin response, developed some dyspnea on exertion and mild cyanosis the evening after the 7^th^ dose of RPQ. The subject was seen in the clinic the following day, and dyspnea had resolved, and the subject was monitored for several days until methemoglobin returned to normal. This reaction did appear to be drug-related; though the subject did not report any exertional dyspnea with SPQ or RSPQ, methemoglobinemia increased prominently during each of these drug arms. This subject was followed by the study nurse and physician until symptoms dissipated (by the next day) and MetHgb returned to normal over several days. She was also examined by a local primary care physician, but no medical intervention was required. This subject was cleared by the physicians to continue in the study.

**TABLE 2:** Adverse events in G6PDn and G6PDd subjects during 7 days oral administration of PQ and its enantiomers.

Parameters	No. Of subjects	Adverse event	Drug-related?	Comments
G6PDn				
Hematological	1/15	• Elevated MetHgB • Dyspnea on exertion • Headache	Yes	Exaggerated MetHgB response to all forms of PQ, symptomatic with RPQ
Liver enzyme markers	2/15	• Increase in AST and ALT	No	• One consumed alcohol during SPQ arm • One subject during placebo arm; found on followup to have history of gallbladder trouble
**G6PDd**				
Hematological	1/2	• Bilirubin increase above preset stop criterion	Yes	Indicative of excess oxidative damage to red cells due to G6PD deficiency

**Note**: All subjects were monitored during the study period and on followup by the study physician until symptoms and signs normalized.

**FIGURE 2:**
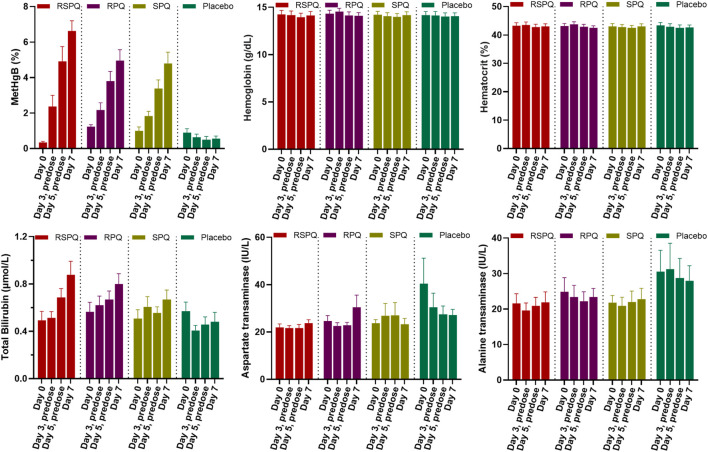
Biochemical and hematological parameters in G6PDn subjects during 7 days daily oral administration of PQ and its individual enantiomers. Bar graphs are presented as mean with SEM (*N* = 15).

In G6PDn subjects, biochemical parameters were not significantly affected except for methemoglobin (MetHgB) and total bilirubin (Figure 2). Significant increases (*p* < .0001) in methemoglobin (MetHgB) from the baseline were seen in subjects receiving PQ enantiomers (at 22.5 mg), although the maximum values averaged about 5% of HgB, and were not associated with clinical symptoms. The increases in MetHgB were not different with the two enantiomers. Subjects receiving racemic primaquine (45 mg) showed a somewhat higher increase in MetHgB, but still only reached a mean of 7% HgB. During placebo administration, MetHgB did not change. From the baseline level, bilirubin progressively increased during daily PQ administration, but these increases were very modest and still under the normal clinical laboratory limits. There were no differences between the enantiomers, and RSPQ showed somewhat higher MetHgB response than the individual enantiomers. In this study, parameters for kidney, liver, and hematological profiles were also analyzed, and no significant changes were observed (Supplementary Tables S1, S2). Findings for vital signs are also tabulated in Supplementary Table S3.

### 3.2 PQ and its metabolite profiling in G6PDn subjects

**FIGURE 3:**
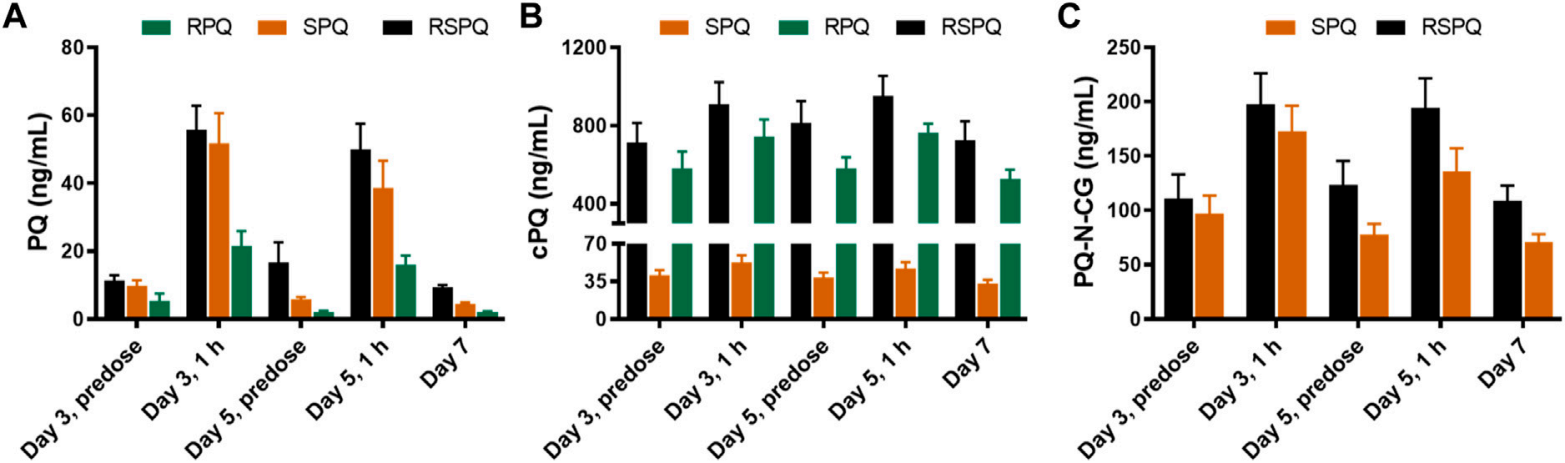
Concentrations of PQ **(A)** and its metabolites **(B,C)** in plasma of G6PDn subjects after oral administration of PQ enantiomers or racemate for 7 days. Bar graphs are presented as mean with SEM (*N* = 15).

During daily PQ administration for 7 days (days 0–6), blood samples were taken on day zero pre-dose, on day three pre-dose and at 1 h post-dose, on day five pre-dose and at 1 h post-dose, and on day seven, 24 h after the last dose. The plasma concentrations of PQ on days 3 and 5 were very low at pre-dose, consistent with our prior study (Khan et al., 2022a) and those of others (Mihaly et al., 1984), and increased 1 h after oral administration (Figure 3). Two major metabolites of PQ (Figure 3A), cPQ (Figure 3B) and PQ-N-CG (Figure 3C), were measured in plasma at these time intervals. When in the SPQ arm, the subjects showed high concentrations of PQ and low concentrations of cPQ; the reverse situation; low PQ and high cPQ was seen during the RPQ arm. When the racemic mixture RSPQ was administered, both PQ and cPQ were high, reflecting the contribution of each enantiomer. Although the dose of RSPQ was the sum of the individual enantiomers, levels of PQ and cPQ were not doubled, presumably reflecting at least in part, a higher apparent volume of distribution (Vd) for RPQ as compared to SPQ in mice (Fasinu et al., 2022), monkeys (Saunders et al., 2014) and humans (Khan et al., 2022a), and also an expected much lower Vd for cPQ as compared to PQ (Baker et al., 1984).

PQ-N-CG, which is formed through direct N-glucuronide conjugation of PQ, was observed only after SPQ administration. Though PQ-N-CG was not detected after RPQ administration, subjects that received RSPQ showed consistently higher plasma levels than those receiving SPQ (Figure 3C).

**FIGURE 4:**
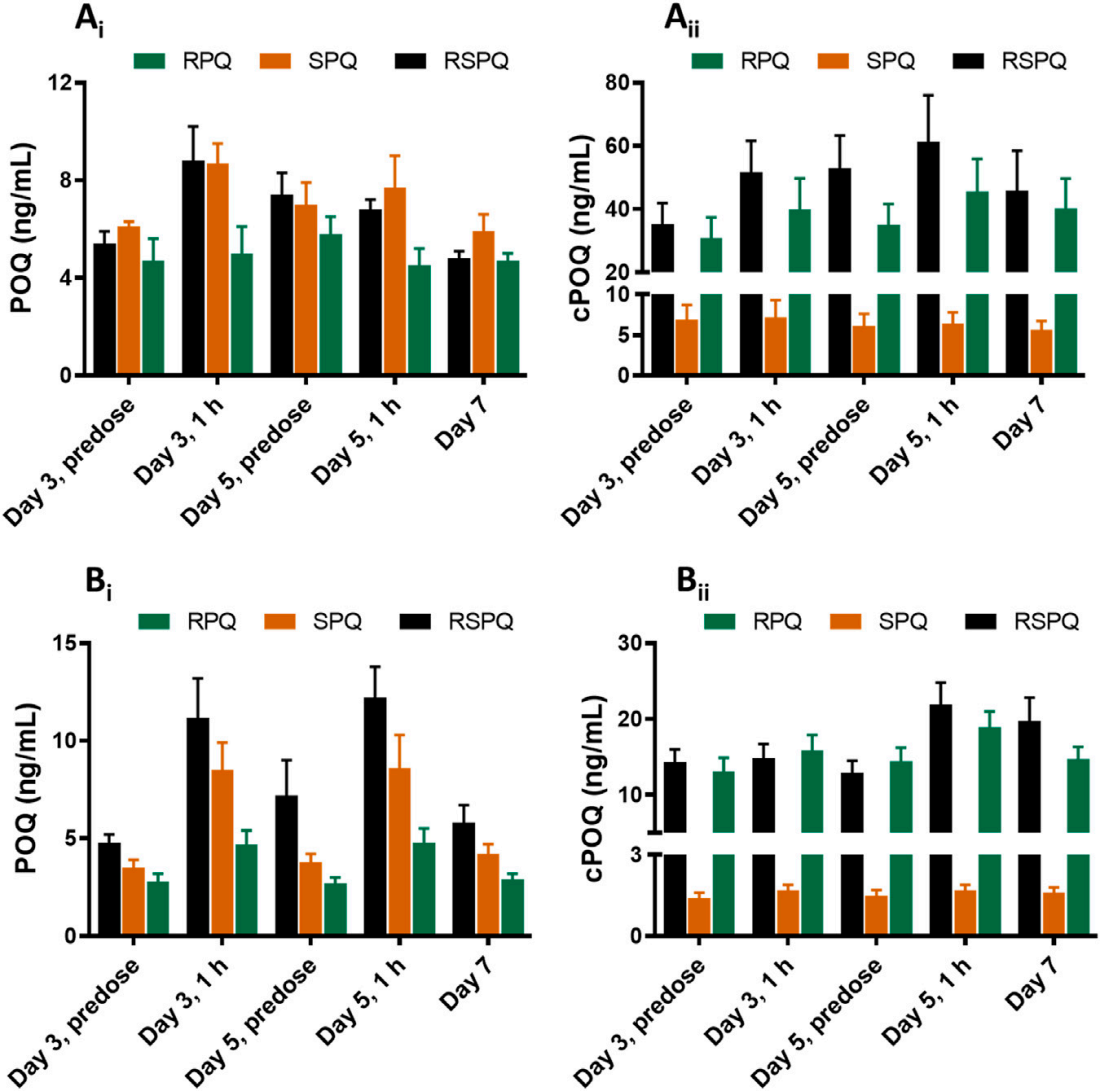
Concentrations of ortho-quinone metabolites of PQ in plasma **(Ai and Aii)** and RBCs **(B**_**i**_ **and B**_**ii**_ **)** of G6PDn subjects during oral administration of PQ enantiomers or racemate for 7 days. Bar graphs are presented as mean with SEM (N = 15).

POQ and cPOQ are the orthoquinones of PQ and cPQ, respectively, formed secondarily to hepatic metabolism, and are believed to reflect the redox-active metabolite pathway for PQ. These two metabolites were measured in plasma and RBCs (Figure 4). POQ was detected in both plasma (Figure 4Ai) and red cells (Figure 4Bi) in the general range of 4–10 ng/ml with an approximate equal distribution between plasma and cells.

POQ levels in plasma were higher in RSPQ and SPQ than in RPQ arm samples as expected due to their higher levels of PQ. cPOQ was found at higher concentrations in plasma (range 30–60 ng/ml) and red cells (range 10–20 ng/ml) in subjects that received RPQ or RSPQ. This is consistent with the higher levels of cPQ in these subjects. Of interest, the concentration of cPOQ in plasma (Figure 4Aii) was notably higher than POQ in all arms of the study; whether this results from enhanced formation or slower elimination of cPOQ is unknown. Similarly, the concentration of cPOQ in RBC (Figure 4Bii) was markedly higher (ca. two-fold) than POQ in all arms, implying that cPQ may contribute more to the oxidant stress within red cells than PQ. Overall, RBC exposure to orthoquinone metabolites showed different profiles depending on which PQ enantiomer is dosed, but both enantiomers contribute to the potential for hemolytic injury.

### 3.3 Findings in G6PDd subjects

**FIGURE 5:**
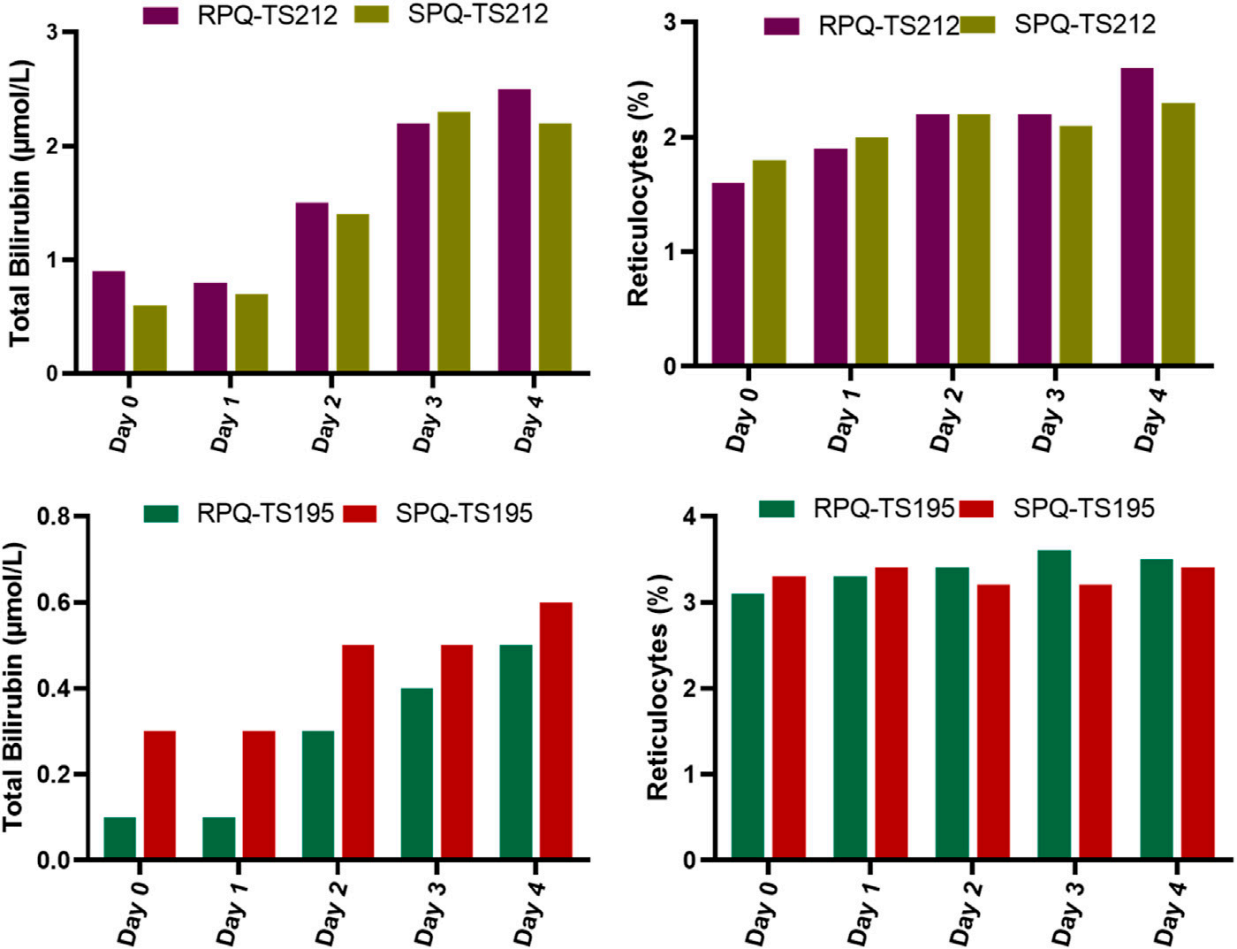
Biochemical and hematological parameters in G6PDd subjects during daily oral administration of the individual enantiomers of PQ.

We were able to enroll two healthy human volunteers with G6PDd in a pilot investigation for 5 days of dosing with the individual PQ enantiomers. One subject was a heterozygous female (A-variant with G6PD activity of 7.4 units/gram of hemoglobin), who received RPQ at a dose of 15 mg daily for 5 days, and after a 2 week washout, received SPQ at the same dose. The other subject was a hemizygous male (A-variant with G6PD activity of 1.2 units/gram of hemoglobin), who was also enrolled to receive both enantiomers. However, this subject (TS212) could not complete the 5 days of dosing with either SPQ or RPQ, since his bilirubin level was significantly increased, and dosing was discontinued after the third dose. Both G6PDd subjects showed progressive significant increase in bilirubin levels with both enantiomers (Figure 5); this was especially more prominent in the hemizygous subject, and reached the stop criterion for discontinuation of dosing after the third dose. Except for lactate dehydrogenase, no other biochemical parameters were changed (Supplementary Tables S4, S5).

**FIGURE 6:**
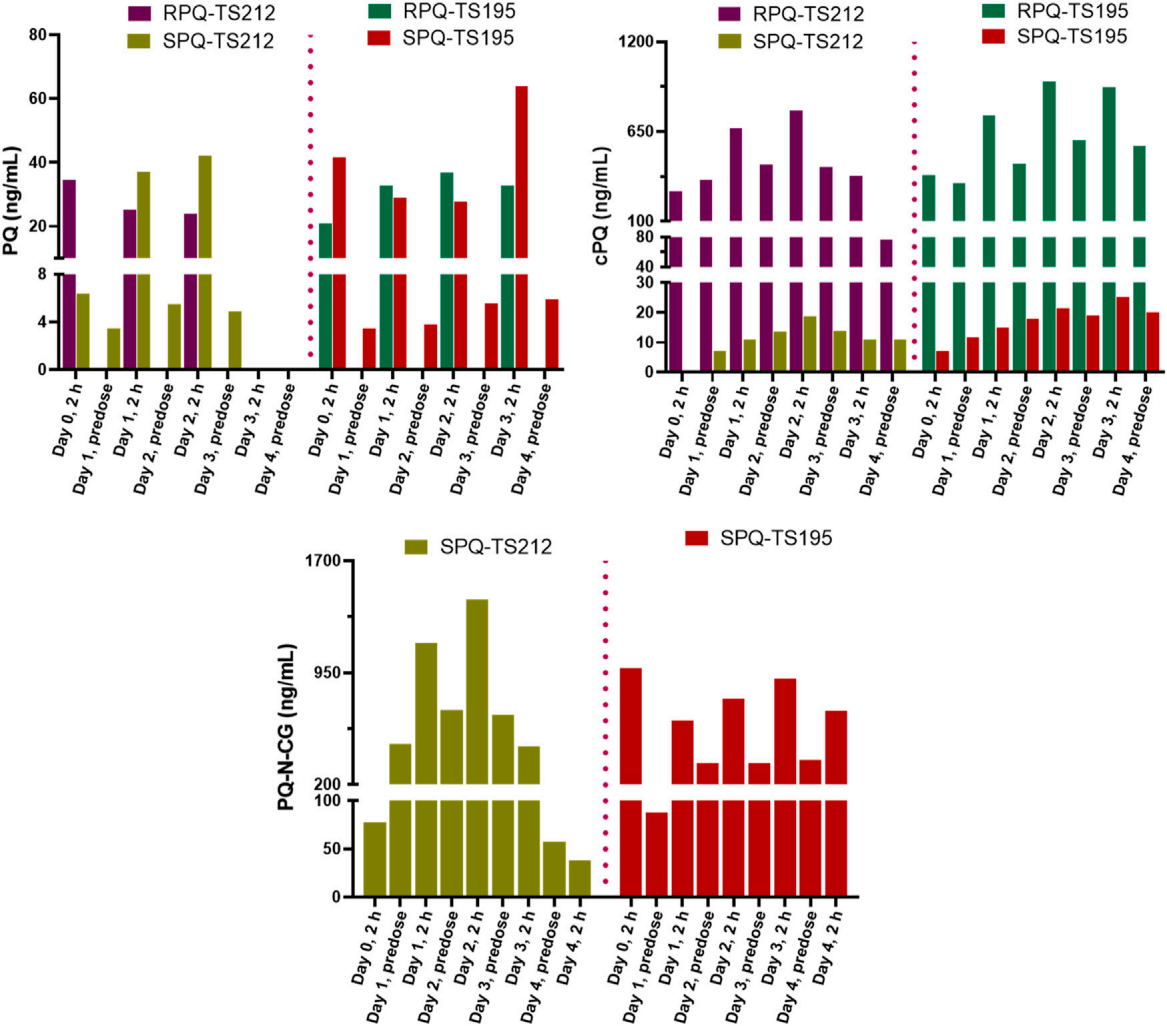
Concentrations of PQ and its metabolite in plasma of G6PDd subjects during daily oral administration of the individual enantiomers of PQ.

**FIGURE 7:**
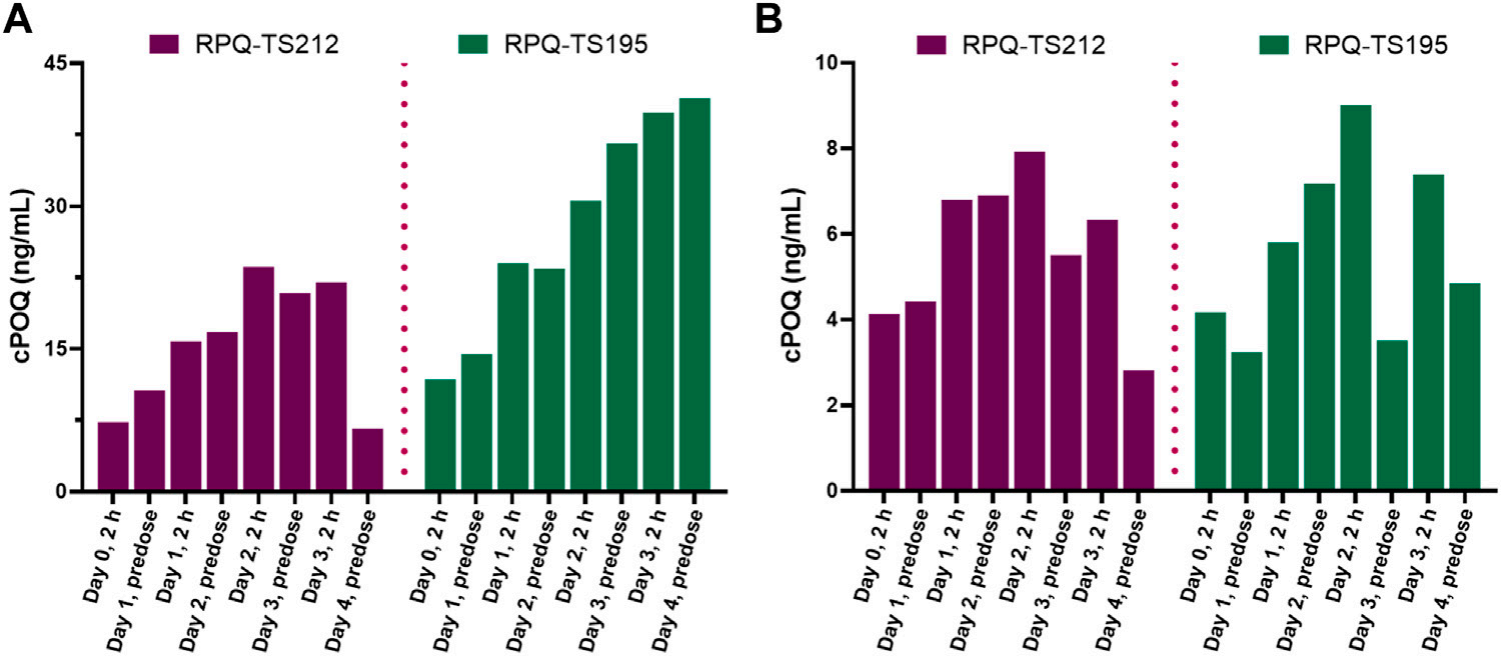
Concentrations of the cPQ metabolite cPOQ in plasma **(A)** and RBCs **(B)** of G6PDd subjects during daily oral administration of the individual enantiomers of PQ.

The G6PDd subjects were closely monitored after dosing, and blood samples were collected at pre-dose and 2 h after administration on days 0, 1, 2, and 3, and then on day 4 (24 h after the last dose). In plasma, PQ, cPQ, PQ-N-CG, and cPOQ were quantified. Like G6PDn subjects, G6PDd subjects also showed a similar pattern of plasma exposure for PQ, cPQ, and PQ-N-CG (Figure 6). Again, PQ exposure in plasma was found to be somewhat higher with SPQ than RPQ, and RPQ showed around 37-fold higher exposure for cPQ than SPQ. POQ could be detected but not quantified in RBCs of G6PDd subjects, likely because of the lower PQ dose. But cPOQ was detectable in plasma and RBCs after RPQ administration (Figure 7A, B), respectively.

## 4 Discussion

The differential pharmacokinetic, metabolism and pharmacologic profiles of individual enantiomers of PQ is now well established, as evidenced in *in vitro* human hepatocytes and also in animals (Fasinu et al., 2014; 2016; 2022; Saunders et al., 2014; Chaurasiya et al., 2021). A recent study from our group also demonstrated highly enantioselective PK and metabolism profiles in subjects treated with single dose of individual PQ enantiomers (Khan et al., 2022a). Since recent clinical studies have suggested the use of a 7 days regimen of PQ for *P. vivax* radical cure (Taylor et al., 2019; Milligan et al., 2020), the current study was undertaken to assess the metabolism of the individual enantiomers of PQ—RPQ and SPQ—and to gauge their general and hematological tolerability in relation to metabolite formation. The rationale for the study was based on the following observations.

### 4.1 PQ metabolism pathways

Although the 8-aminoquinoline class of antimalarials has an unique place in malaria chemotherapy, their optimal employment is limited due to safety concerns in individuals with G6PDd (Clyde, 1981; Baird and Rieckmann, 2003). It is established that this liability in G6PDd is due to the enhanced sensitivity of their red cells to oxidative stressors. In the case of PQ, this has long been attributed to redox active metabolites (Allahyari et al., 1984; Bowman et al., 2004; Ganesan et al., 2009; 2012), though the precise nature of the metabolites and their pathways/locations *in vivo* were for many years uncertain (Strother et al., 1984; Marcsisin et al., 2016; Flaherty et al., 2022). cPQ is well known as a major circulating metabolite, likely formed by the actions of monoamine oxidases (Nicholl et al., 1987; Fasinu et al., 2016). cPQ is not redox active. Other pathways are also now well established, including the hydroxylation of PQ by cytochrome P450 (CYP) pathways, observed using isolated enzyme systems, liver microsomes, and in intact human hepatocytes (Avula et al., 2013; Jin et al., 2014; Ariffin et al., 2019). Among these CYPs, CYP 2D6 accomplishes the formation of at least four different metabolites *via* hydroxylation of the PQ ring at 2-, 3-, four- and five- positions (Pybus et al., 2012; Fasinu et al., 2014; Potter et al., 2015). This CYP 2D6 activity has been shown to be an important determinant of the altered metabolism of PQ in animals (Pybus et al., 2013) and humans (Potter et al., 2015), and poor CYP 2D6 metabolizer phenotypes show a reduced anti-relapse efficacy of PQ (Bennett et al., 2013). These hydroxylated metabolites have proven difficult to detect in plasma, whether by virtue of instability, compartmentalization within tissues, or rapid conjugation and excretion (Mihaly et al., 1984).

Several studies have now shown that 5-OH-PQ is exceedingly unstable, rapidly oxidizing to its quinone-imine, then to 5,6-dihydroxy-PQ, and finally to the 5,6-orthoquinone (POQ) (Camarda et al., 2019; Fasinu et al., 2019). Even the more stable POQ is not typically observed in plasma, though it is detected readily in urine (Spring et al., 2019).

Recently, our group also reported that POQ can be formed directly from PQ on incubation with human red cells, presumably *via* heme- or hemoglobin-dependent formation of oxygen free radicals (Fasinu et al., 2019); the qualitative importance of this *in situ* generation of oxidants vis-a-vis that derived from hepatic metabolism is yet to be determined. We have also observed that cPQ can also give rise to an ortho-quinone type metabolite (cPOQ), presumably by analogous metabolic routes, but this does not appear to be accomplished by CYP 2D6 (Khan et al., 2021). The relative contributions of POQ and cPOQ to red cell oxidant stress and subsequent injury in humans is clearly of interest to resolution of the importance of the two enantiomers in the clinical use of PQ.

### 4.2 Stereochemistry and pharmacodynamics and metabolism of PQ

PQ is a racemic mixture of (*R*) and (*S*) enantiomers, and our prior studies have shown vastly different metabolism of the two enantiomers, whether with *in vitro* drug metabolizing enzymes (Pybus et al., 2012; Fasinu et al., 2014; 2016), in animal models (Baker and McChesney, 1988; Saunders et al., 2014), or in humans (Tekwani et al., 2015; Khan et al., 2022a). RPQ is much more significantly converted *via* the MAO pathway to cPQ, the major PQ metabolite in human plasma. PQ-N-CG has been recently identified as a PQ metabolite in plasma, notably after administration of SPQ (Khan et al., 2022a). The formation of the hydroxylated products of PQ also showed quite distinct patterns with the two enantiomers, though both RPQ and SPQ gave rise to all of the reported ring-hydroxylated species (Fasinu et al., 2016). Several reported animal studies have shown dramatic differences in the toxicity of PQ enantiomers, including their hematological effects, with SPQ showing greater systemic toxicity than RPQ in mice (Schmidt et al., 1977), but lower hepatotoxicity in primates (Schmidt et al., 1977; Saunders et al., 2014); however, hematological effects of SPQ were more pronounced than RPQ’s effects in both species (Nanayakkara et al., 2014; Saunders et al., 2014); this is in spite of an equivalent radical curative efficacy of the two enantiomers in primates (Schmidt et al., 1977; Saunders et al., 2014).

A critical question that arises, then, for the safe use of PQ in humans with G6PDd is whether the differential metabolic profile of enantiomers will translate to a better therapeutic index. Toward answering that question, we undertook to assess the tolerability and metabolism of the RPQ and SPQ, first in G6PDn human volunteers receiving a single dose (Khan et al., 2022a). SPQ showed higher plasma exposure of PQ as compared to RPQ. cPQ was the predominant plasma metabolite with RPQ administration, many folds higher than the parent drug, but PQ-N-CG was not detected. In contrast, for SPQ, PQ-N-CG was the major plasma metabolite, while much lower levels of cPQ were seen (Khan et al., 2022a). POQ and cPOQ were also identified in the RBCs of subjects receiving single doses (Khan et al., 2021). However, in this study using single doses, there were no identifiable tolerability issues, so we could not distinguish any safety advantage. Typically, the hematological toxicity of PQ in G6PDd subjects appear after a few days dosing.

### 4.3 The current study

In the current study we aimed to check the comparative tolerability of racemic PQ and its individual enantiomers in G6PDn subjects, and profile the plasma and RBCs for parent and metabolites during oral administration once daily for seven consecutive days. Two G6PDd individuals also received PQ enantiomers for a 5 day course.

### 4.4 PQ metabolism in G6PDn

With respect to metabolism, as observed in our single dose study (Khan et al., 2022a), plasma exposure to parent drug was significantly higher for SPQ treated groups than for RPQ, and RSPQ behaved in an additive fashion. Plasma cPQ levels were much higher after RPQ administration than SPQ, likely reflecting the greater vulnerability of RPQ to metabolism by monoamine oxidases (Chaurasiya et al., 2021). This presumably also accounts for the reduced exposure to the parent drug after RPQ administration. Of note, cPQ concentrations were sustained in the blood over the 24 h daily dosing interval, such that it was still very high when subsequent doses were administered. Consistent with this prolonged exposure, higher and sustained levels of cPOQ were observed in plasma and RBC, as compared with POQ, raising the possibility that cPQ may contribute more to the overall oxidant stress and haemolytic activity seen clinically than PQ does directly. If so, and if the two enantiomers show similar redox cycling within the red cell, it would follow that the RPQ enantiomer would contribute more to the hemotoxicity of the parent drug than the SPQ enantiomer, with implications for difference in therapeutic index. Conversely, if the sustained higher levels of cPOQ in red cells is due to lesser capacity to redox cycle, and hence lesser production of active oxygen species, RPQ may have carry less potential for haemolytic injury.

In addition, SPQ is cleared by metabolism to PQ-N-CG *via* a phase II conjugation pathway. N-carbamoylation and glucuronidation of amines is relatively uncommon, but not unknown (Obach et al., 2006). The analogous metabolite of RPQ was not observed in this study or in our previous single dose study (Khan et al., 2022a). The contribution of this metabolite to the therapeutic efficacy of primaquine is not known, but a possible role implies an additional further differentiation in the therapeutic index of the two enantiomers.

Collectively, these considerations further emphasize the relevance of the stereoselective metabolism of primaquine in the clinical use of this drug.

The orthoquinone metabolite of PQ, derived from 5-hydroxylation (POQ) was observed only at very low concentrations in circulation after PQ administration. Further, POQ concentrations in red cells after SPQ administration were higher than after RPQ, and RSPQ showed an additive response. In other words, POQ in red cells appeared to fluctuate in a manner reflecting PQ plasma concentrations.

Our findings with respect to PQ metabolism in the two G6PDd subjects (one heterozygous female, one hemizygous male) did not show any clear differences from the G6PDn subjects with respect to the metabolic clearance of the enantiomers. These data further support the concept that the greater susceptibility of G6PDd individuals to hemolytic anemia after PQ lies in the lesser protective ability of their red cells and not in enhanced production of oxidant metabolites.

Even though the POQ in RBCs after SPQ is somewhat higher than after RPQ, the latter gives rise to substantially higher amounts of cPOQ in both plasma and RBCs. Although POQ has long been assumed to reflect the redox active pathways for efficacy and toxicity of PQ, any contribution of cPOQ in this respect has not been anticipated.

### 4.5 Tolerability/safety

Both PQ enantiomers were well tolerated in G6PDn individuals with 7 day dosing at 22.5 mg (or with 45 mg of RSPQ). The primary findings of interest here were the progressive elevations of methemoglobin (MetHgB) and total bilirubin observed. MetHgB is formed on oxidation of hemoglobin when the ferrous ion is converted to ferric (Rifkind et al., 2015). Methemoglobinemia is one of the hallmark effects of PQ with multiple days dosing. However, methemoglobinemia, *per se*, is rarely of serious clinical significance with PQ administration (Carmona-Fonseca et al., 2009). A rise in MetHgB was observed in most of our subjects, and there was no difference between the two enantiomers. RSPQ tended to show a higher response, as the dose was double that with the racemate, but in only one subject—the most sensitive responder—was a clinical impact observed. This subject showed some transient exertional dyspnea on the RSPQ arm that required discontinuation of dosing after the 5^th^ dose. However, this subject responded to each drug form—RPQ, SPQ and RSPQ with the most robust MetHgB rise of any subject—to above 10% of HgB with each enantiomer and with the racemate. This subject did not show any other hematological or biochemical abnormalities on study endpoints, but displayed an elevated thyroid stimulating hormone (TSH). All other subjects exhibited minimal to modest responses with a gradual rise in MetHgB to an average of 4%–5% of HgB. Our findings are thus consistent with earlier reports that methemoglobinemia is generally not a limiting concern with PQ administration, in that many subjects experience mild increases, with no obvious impact on hemolysis (Carmona-Fonseca et al., 2009).

This study was not designed to address gender differences—nor was it sufficiently powered for such. However, safety parameters and profiles of PQ and metabolites were considered to assess any obvious distinctions. In terms of safety, there did not appear to be anything thing clinically significant. The mean MetHgB response in females is somewhat greater than in males after RSPQ administration, but this seems largely influenced by the one female subject mentioned earlier with an exaggerated response. We did find a significantly increased POQ content in red cells in female subjects, as compared to males. These data are depicted in the supplemental files (Supplementary Figures S1, S2). The significance of this—if any—is unknown, but may be a subject for exploration as further field studies with PQ are pursued.

A somewhat unexpected finding of this study was the sensitivity of the total bilirubin responses in G6PDn subjects. Though the changes were still well below the clinical upper limits of normal and did not approach clinical significance in terms of patient safety, the gradual increase with daily dosing was observed consistently. Placebo administration did not result in any increase in bilirubin. In one subject with severe G6PDd deficiency, the rise in bilirubin was much exaggerated, even with a lower dose (15 mg daily) of either of the enantiomers, such that the preset safety limits were exceeded, and dosing had to be discontinued after 3 days with both RPQ and SPQ. The heterozygous female subject showed enhanced responses to the G6PDn individuals in terms of bilirubin. The increase in total bilirubin after PQ or its enantiomers were confirmed to be due to indirect bilirubin (unconjugated), and thus likely derived from red cell destruction, rather than liver injury. This suggests that a minor fraction of red cells—perhaps the oldest fraction in circulation, which display the cumulative oxidative insults of normal cell aging—are impacted by the addition of the oxidative stress of the PQ metabolites. Early studies of PQ sensitivity suggested that even in G6PDn subjects, subclinical responses in various endpoints can be observed, likely due to a minor fraction of aging red cells with lower G6PD and other glycolytic enzymes (Powell and Degowin, 1965). The changes in bilirubin were not significantly different between RPQ and SPQ, though there was a slight tendency toward a greater RPQ response. Our studies suggest that bilirubin might serve as a better hemolysis-relevant marker in G6PDn individuals as compared to methemoglobin.

## 5 Conclusion

Studies herein indicate that a course of seven daily doses of racemic primaquine (45 mg) or its individual enantiomers (at 22.5 mg), are very well tolerated in G6PDn individuals, with no apparent differences in safety of the enantiomers, in spite of widely divergent exposure to the parent drug and key metabolites. Gradual increases in methemoglobin and bilirubin were consistently observed with both enantiomers and racemate. The bilirubin response in a single hemizygous G6PDd male was much exaggerated, and required discontinuation with both enantiomers.

## Data Availability

The original contributions presented in the study are included in the article/supplementary material, further inquiries can be directed to the corresponding authors.
