# Supplementary material for: Comparative metabolism and tolerability of racemic primaquine and its enantiomers in human volunteers during 7-day administration
Source: Front Pharmacol. 2023 Jan 16;13:1104735. doi: 10.3389/fphar.2022.1104735 (PMC9885159; doi:10.3389/fphar.2022.1104735)
Supplement: Supplementary file 1 [file Table1.docx]

**Supplementary File**

[1. Biochemical parameters of G6PDn subjects 2](#_Toc124102050)

[Table S1: Biochemical parameters of G6PDn subjects. 2](#_Toc124102051)

[Table S2: Hematological parameters of G6PDn subjects. 3](#_Toc124102052)

[Table S3: Vital signs of G6PDn subjects. 4](#_Toc124102053)

[2. Evaluation of Male and Female subjects’ responses based on their analyte profile in plasma and RBCs. 5](#_Toc124102054)

[Figure S1: RBCs profile of POQ in G6PDn male (N=11) and female (N=4) subjects during 7-d daily oral administration of PQ and its individual enantiomers. Data are presented as Mean with SEM. 5](#_Toc124102055)

[Figure S2: Plasma profile of PQ in G6PDn male (N=11) and female (N=4) subjects during 7-d daily oral administration of PQ and its individual enantiomers. Data are presented as Mean with SEM. 6](#_Toc124102056)

[3. Biochemical parameters of G6PDd subjects 7](#_Toc124102057)

[Table S4: Biochemical parameters of G6PDd subject (TS212). 7](#_Toc124102058)

[Table S5: Biochemical parameters of G6PDd subject (TS195). 8](#_Toc124102059)

# Biochemical parameters of G6PDn subjects

## Table S1: Biochemical parameters of G6PDn subjects.

| **Biochemical parameters** | **RSPQ** | **RPQ** | **SPQ** | **Placebo** |
| --- | --- | --- | --- | --- |
| **Alkaline Phosphatase (IU/L)** | | | | |
| Day 0 | 62.1 ± 3.5 | 64.9 ± 3.7 | 64.0 ± 3.5 | 66.0 ± 3.4 |
| Day 3, predose | 63.1 ± 3.3 | 65.2 ± 3.5 | 65.8 ± 3.8 | 63.2 ± 3.4 |
| Day 5, predose | 61.1 ± 3.2 | 64.3 ± 4.1 | 63.2 ± 3.4 | 62.6 ± 3.4 |
| Day 7 | 63.0 ± 3.6 | 65.0 ± 4.0 | 66.4 ± 3.7 | 65.2 ± 3.7 |
| **Blood urea nitrogen (mg/dL)** | | | | |
| Day 0 | 12.6 ± 0.7 | 13.6 ± 1.2 | 12.5 ± 0.9 | 12.8 ± 0.8 |
| Day 3, predose | 12.3 ± 0.8 | 12.7 ± 0.7 | 13.2 ± 1.1 | 12.6 ± 0.6 |
| Day 5, predose | 10.9 ± 0.7 | 13.4 ± 0.9 | 13.1 ± 0.8 | 12.5 ± 0.8 |
| Day 7 | 12.0 ± 0.8 | 12.5 ± 0.9 | 11.5 ± 0.8 | 12.4 ± 0.6 |
| **Creatinine (mg/dL)** | | | | |
| Day 0 | 0.9 ± 0.05 | 0.9 ± 0.04 | 0.9 ± 0.04 | 0.9 ± 0.04 |
| Day 3, predose | 0.9 ± 0.05 | 0.9 ± 0.04 | 0.9 ± 0.04 | 0.9 ± 0.05 |
| Day 5, predose | 0.9 ± 0.04 | 0.9 ± 0.04 | 0.9 ± 0.04 | 0.9 ± 0.04 |
| Day 7 | 0.9 ± 0.05 | 0.9 ± 0.04 | 0.9 ± 0.03 | 0.9 ± 0.05 |
| **Albumin (g/dL)** | | | | |
| Day 0 | 4.5 ± 0.08 | 4.6 ± 0.05 | 4.5 ± 0.07 | 4.6 ± 0.08 |
| Day 3, predose | 4.5 ± 0.08 | 4.6 ± 0.07 | 4.5 ± 0.07 | 4.5 ± 0.10 |
| Day 5, predose | 4.6 ± 0.05 | 4.5 ± 0.08 | 4.5 ± 0.09 | 4.5 ± 0.08 |
| Day 7 | 4.6 ± 0.08 | 4.6 ± 0.07 | 4.6 ± 0.07 | 4.6 ± 0.07 |
| **Lactate dehydrogenase** | | | | |
| Day 0 | 156.8 ± 4.1 | 169.5 ± 4.8 | 178.5 ± 4.5 | 156.8 ± 5.4 |
| Day 3, predose | 156.6 ± 4.5 | 157.7 ± 5.0 | 160.9 ± 7.6 | 157.4 ± 7.3 |
| Day 5, predose | 155.4 ± 6.0 | 156.7 ± 4.0 | 171.2 ± 11.7 | 154.1 ± 4.6 |
| Day 7 | 161.6 ± 5.7 | 174.4 ± 15.2 | 159.0 ± 4.9 | 158.8 ± 5.1 |

Data are presented as Mean with SEM (N = 15)

## Table S2: Hematological parameters of G6PDn subjects.

| **Hematological parameters** | **RSPQ** | **RPQ** | **SPQ** | **Placebo** |
| --- | --- | --- | --- | --- |
| **WBC (X10E^3^/µL)** | | | | |
| Day 0 | 5.8 ± 0.4 | 5.8 ± 0.5 | 5.8 ± 0.6 | 6.0 ± 0.4 |
| Day 3, predose | 5.9 ± 0.3 | 6.1 ± 0.5 | 5.9 ± 0.4 | 5.9 ± 0.4 |
| Day 5, predose | 6.1 ± 0.6 | 5.7 ± 0.5 | 5.7 ± 0.4 | 5.7 ± 0.4 |
| Day 7 | 6.0 ± 0.3 | 5.4 ± 0.4 | 5.3 ± 0.5 | 6.0 ± 0.5 |
| **RBC (X10E^6^/µL)** | | | | |
| Day 0 | 4.9 ± 0.1 | 4.9 ± 0.1 | 4.8 ± 0.1 | 4.8 ± 0.1 |
| Day 3, predose | 4.9 ± 0.1 | 4.9 ± 0.1 | 4.8 ± 0.1 | 4.8 ± 0.1 |
| Day 5, predose | 4.8 ± 0.1 | 4.8 ± 0.1 | 4.7 ± 0.1 | 4.7 ± 0.1 |
| Day 7 | 4.8 ± 0.1 | 4.8 ± 0.1 | 4.8 ± 0.1 | 4.8 ± 0.1 |
| **Platelets (X10E^3^/µL)** | | | | |
| Day 0 | 236.6 ± 11.7 | 249.1 ± 15.0 | 253.0 ± 13.4 | 251.1 ± 13.2 |
| Day 3, predose | 235.1 ± 11.6 | 248.3 ± 12.8 | 242.4 ± 13.2 | 243.6 ± 13.2 |
| Day 5, predose | 227.7 ± 11.0 | 249.1 ± 13.0 | 234.6 ± 12.9 | 247.6 ± 12.7 |
| Day 7 | 230.1 ± 11.2 | 255.3 ± 11.5 | 242.3 ± 13.9 | 251.9 ± 13.2 |
| **HptGb** | | | | |
| Day 0 | - | 89.6 ± 10.7 | - | - |
| Day 3, predose | 87.4 ± 10.9 | 97.0 ± 11.5 | 97.7 ± 13.2 | 97.1 ± 10.5 |
| Day 5, predose | 84.2 ± 10.2 | 86.2 ± 11.2 | 95.9 ± 11.8 | 87.5 ± 9.1 |
| Day 7 | 85.2 ± 10.4 | 86.0 ± 11.9 | 92.1 ± 11.5 | 89.7 ± 9.69 |

Data are presented as Mean with SEM (N = 15)

## Table S3: Vital signs of G6PDn subjects.

| **Vital signs** | **RSPQ** | **RPQ** | **SPQ** | **Placebo** |
| --- | --- | --- | --- | --- |
| **Systolic BP** | | | | |
| Day 0 | 111.5 ± 4.5 | 116.1 ± 3.5 | 115.2 ± 2.7 | 105.5 ± 7.8 |
| Day 3, predose | 112.7 ± 3.2 | 109.4 ± 6.1 | 113.2 ± 1.9 | 105.3 ± 7.9 |
| Day 5, predose | 105.9 ± 2.4 | 111.2 ± 2.2 | 113.3 ± 2.9 | 110.1 ± 3.8 |
| Day 7 | 112.0 ± 2.8 | 111.5 ± 2.7 | 112.5 ± 3.1 | 116.5 ± 2.7 |
| **Diastolic BP** | | | | |
| Day 0 | 71.6 ± 2.6 | 75.6 ± 1.9 | 75.1 ± 1.8 | 74.3 ± 0.4 |
| Day 3, predose | 73.2 ± 1.5 | 74.1 ± 1.5 | 72.2 ± 1.1 | 73.6 ± 0.1 |
| Day 5, predose | 67.1 ± 1.4 | 71.5 ± 1.8 | 72.8 ± 1.7 | 73.8 ± 0.1 |
| Day 7 | 70.5 ± 1.9 | 72.4 ± 1.4 | 70.1 ± 1.9 | 75.6 ± 0.1 |
| **Pulse** | | | | |
| Day 0 | 74.3 ± 4.2 | 81.1 ± 3.8 | 71.3 ± 5.1 | 82.3 ± 4.1 |
| Day 3, predose | 72.5 ± 2.1 | 76.6 ± 3.7 | 72.3 ± 2.9 | 73.8 ± 3.5 |
| Day 5, predose | 80.8 ± 3.4 | 77.1 ± 2.8 | 76.7 ± 3.2 | 76.1 ± 0.2 |
| Day 7 | 83.8 ± 3.4 | 78.2 ± 2.9 | 76.1 ± 3.5 | 81.6 ± 3.4 |
| **Resp rate** | | | | |
| Day 0 | 20.0 ± 0.0 | 20.0 ± 0.2 | 19.8 ± 0.1 | 19.3 ± 0.1 |
| Day 3, predose | 19.9 ± 0.1 | 20.0 ± 0.2 | 19.3 ± 0.2 | 19.6 ± 0.1 |
| Day 5, predose | 19.5 ± 0.2 | 19.7 ± 0.2 | 19.3 ± 0.3 | 19.8 ± 0.1 |
| Day 7 | 19.6 ± 0.2 | 20.1 ± 0.1 | 19.8 ± 0.1 | 19.8 ± 0.1 |
| **SPO2%** | | | | |
| Day 0 | 98.1 ± 0.2 | 98.1 ± 0.3 | 97.6 ± 0.3 | 98.1 ± 0.2 |
| Day 3, predose | 96.1 ± 0.4 | 97.3 ± 0.4 | 96.9 ± 0.3 | 97.2 ± 0.3 |
| Day 5, predose | 94.8 ± 0.3 | 96.4 ± 0.4 | 96.6 ± 0.3 | 97.6 ± 0.3 |
| Day 7 | 94.2 ± 0.5 | 96.3 ± 0.3 | 96.0 ± 0.5 | 97.4 ± 0.4 |

Data are presented as Mean with SEM (N = 15)

# Evaluation of Male and Female subjects’ responses based on their analyte profile in plasma and RBCs.

## Figure S1: RBCs profile of POQ in G6PDn male (N=11) and female (N=4) subjects during 7-d daily oral administration of PQ and its individual enantiomers. Data are presented as Mean with SEM.

## Figure S2: Plasma profile of PQ in G6PDn male (N=11) and female (N=4) subjects during 7-d daily oral administration of PQ and its individual enantiomers. Data are presented as Mean with SEM.

# Biochemical parameters of G6PDd subjects

## Table S4: Biochemical parameters of G6PDd subject (TS212).

| **Biochemical parameters** | **RPQ** | **SPQ** |
| --- | --- | --- |
| **HGB (g/dL)** | | |
| Day 0 | 12.4 | 12.5 |
| Day 1 | 12.4 | 13.1 |
| Day 2 | 13.0 | 13.2 |
| Day 3 | 12.2 | 12.7 |
| Day 4 | 11.5 | 12.2 |
| **HCT (%)** | | |
| Day 0 | 39.5 | 40.0 |
| Day 1 | 39.4 | 42.0 |
| Day 2 | 40.9 | 41.0 |
| Day 3 | 38.1 | 40.6 |
| Day 4 | 36.7 | 40.2 |
| **HAPT (mg/dL)** | | |
| Day 0 | 208.0 | 156.0 |
| Day 1 | 215.0 | 163.0 |
| Day 2 | 224.0 | 163.0 |
| Day 3 | 195.0 | 161.0 |
| Day 4 | 175.0 | 157.0 |
| **LDH (U/L)** | | |
| Day 0 | 179 | 161 |
| Day 1 | 168 | 168 |
| Day 2 | 244 | 175 |
| Day 3 | 186 | 171 |
| Day 4 | 159 | 165 |

## Table S5: Biochemical parameters of G6PDd subject (TS195).

| **Biochemical parameters** | **RPQ** | **SPQ** |
| --- | --- | --- |
| **HGB (g/dL)** | | |
| Day 0 | 12.5 | 13.3 |
| Day 1 | 12.6 | 13.2 |
| Day 2 | 12.7 | 12.7 |
| Day 3 | 12.0 | 12.3 |
| Day 4 | 12.8 | 12.0 |
| **HCT (%)** | | |
| Day 0 | 38.6 | 42.2 |
| Day 1 | 38.8 | 41.0 |
| Day 2 | 39.1 | 38.9 |
| Day 3 | 38.5 | 38.8 |
| Day 4 | 39.5 | 40.0 |
| **HAPT (mg/dL)** | | |
| Day 0 | 96 | 132 |
| Day 1 | 89 | 132 |
| Day 2 | 96 | 128 |
| Day 3 | 96 | 126 |
| Day 4 | 97 | 133 |
| **LDH (U/L)** | | |
| Day 0 | 143 | 166 |
| Day 1 | 468 | 179 |
| Day 2 | 147 | 172 |
| Day 3 | 166 | 390 |
| Day 4 | 175 | 226 |
